# Bromelia pinguin Extract Mitigates Glyphosate-Induced Toxicity in Human Cells

**DOI:** 10.7759/cureus.74701

**Published:** 2024-11-28

**Authors:** Luis Omar Masías-Ambriz, Mario Daniel Caba-Flores, Nereida Montes-Castro, Israel García-Aguiar, Ruben Ruiz-Ramos, Edgar Zenteno, Carmen Martínez-Valenzuela

**Affiliations:** 1 Genotoxicology Laboratory, Universidad Autónoma de Occidente, Los Mochis, MEX; 2 Biochemistry and Molecular Medicine, Universidad Autónoma de Nuevo León, Monterrey, MEX; 3 Biomedical Sciences, Universidad Autónoma de Occidente, Culiacán, MEX; 4 Molecular Toxicology Laboratory, Universidad Veracruzana, Veracruz, MEX; 5 Biochemistry Laboratory, Universidad Nacional Autónoma de México, Ciudad de México, MEX

**Keywords:** anti-inflammatory, apoptosis, bromelia pingüin, caspase-1, cytotoxicity, glyphosate, nlrp3, parp-1, pbmc

## Abstract

Introduction: Extensive agricultural activity results in significant exposure to pesticides, particularly glyphosate, which has been linked to immunological disorders, including apoptosis and inflammation. *Bromelia pinguin*, a species from the Bromeliaceaefamily native to Mexico, is traditionally used in folk medicine for its medicinal properties, including anti-inflammatory effects. This research aimed to evaluate the protective effects of *Bromelia pinguin *extract on human peripheral blood mononuclear cells (PBMCs) exposed to Faena®, a commercially available glyphosate-based herbicide.

Methods: PBMCs were isolated from healthy donors. Cells were exposed to varying concentrations of glyphosate commercial formulation Faena®, pure potassium glyphosate salts, and *Bromelia pinguin *extract alone and in co-exposure studies with the extract. Dose-response curves were performed to determine IC_50_. Cell viability was assessed, and the expression of inflammatory and apoptotic markers, including Caspase-1, NLRP3, and PARP-1, was analyzed.

Results: Exposure of PBMCs to glyphosate salts and Faena® resulted in a dose-dependent reduction of cell viability, with IC_50_ values of 669.376 µg/mL and 6.555 µg/mL, respectively. Co-exposure of cells with *Bromelia pinguin*, extract significantly improved cell viability up to 25% in both herbicide-treated groups. Western blot analysis revealed increased levels of Caspase-1, NLRP3, and PARP-1 after herbicide exposure, indicating activation of apoptotic and inflammatory pathways. Treatment with *Bromelia pinguin*, extract mitigated the expression of these markers.

Conclusion: The extract of *Bromelia pinguin *can enhance cell viability and reduce the upregulation of inflammatory and apoptotic markers in human PBMCs exposed to glyphosate-based herbicides. These results provide new insights into the therapeutic potential of plant-based interventions in pesticide-induced immunological and inflammatory problems*.*

## Introduction

Global population growth demands increased food production, making crops vital for both humans and livestock. However, crops are susceptible to diseases, pests, and undesirable weeds, leading to the widespread use of pesticides to improve crop quality and yields [1,2]. Among more than 100 classes of pesticides, glyphosate-based formulations are the most commonly used worldwide for weed control [2,3]. However, the growing use of glyphosate in agricultural practices has raised significant concerns due to its environmental impact, persistence, and, importantly, its potential toxicity and negative impact on human health [2,4]. Occupational exposure to glyphosate is associated with acute health risks [2,5], while the general population faces chronic exposure through environmental pollution and continuous ingestion of contaminated food and water [2,6]. This chronic exposure can increase populations living near zones with intense agricultural activity [7]. Health traits linked to glyphosate include cancer risk, endocrine disruption, gastrointestinal and respiratory issues, hepatorenal damage, and apoptotic and inflammatory disruptive effects [6,8,9]. Unfortunately, there is currently no specific antidote for glyphosate exposure, and treatment remains symptomatic [10].

In this context, natural products with anti-inflammatory properties have gained interest as potential therapeutic alternatives. Extracts from plants of the Bromeliaceae family have demonstrated promising anti-inflammatory effects in vivo, showing efficacy in both acute and chronic inflammation models [11]. One study demonstrated the anti-inflammatory effects through in vitro studies with human leukocytes, and in vivo studies confirmed a significant decrease in neutrophil migration to acute inflammation sites [12]. These findings suggest the potential benefits of plant extracts to mitigate inflammatory effects induced by glyphosate pesticides, however to date such studies are lacking.

*Bromelia pinguin* is a plant native to Latin America and the Caribbean islands. It is widely used in traditional medicine and has shown promising bioactive effects, including antifungal [13] and antiparasitic activities [14]. However, its anti-inflammatory and antiapoptotic effects have not been evaluated.

This study aims to evaluate the in vitro anti-inflammatory and anti-apoptotic effects of aqueous extracts of *Bromelia pinguin* on peripheral blood mononuclear cells (PBMCs) exposed to glyphosate salts and the commercially available glyphosate-based herbicide Faena®. The goal is to explore the potential protective effects of this plant extract that is widely available in northern Mexico, and its implications for developing natural, sustainable interventions to counteract the toxic effects of widely used herbicides like glyphosate in global agricultural practices, particularly in regions with intense agricultural activities like the northern Mexican state of Sinaloa.

## Materials and methods

Study design

Peripheral blood mononuclear cells (PBMCs) were isolated and exposed to either pure glyphosate potassium salts or a commercially available glyphosate formulation, Faena® Clásico (Monsanto ®). Following herbicide treatments and co-exposure with the plant extract, cell viability was determined by exclusion analysis using trypan blue exclusion analysis, and the expression of inflammatory and apoptotic markers such as Caspase-1, NLRP3, and PARP-1 was evaluated through Western blot.

Participant recruitment

This study was conducted between 2021 and 2023. PBMCs were obtained from three healthy male adult volunteers aged 20-23 years old, with no previous exposure to herbicides, and no chronic diseases, tobacco, alcohol or illicit drug use.

Blood collection and PBMCs isolation

Peripheral blood samples (10 mL) were collected in Ethylenediamine tetraacetic acid (EDTA) tubes from healthy adult volunteers. For PBMC isolation, 1 ml of blood was mixed with 2 mL of Ficoll-Paque in a 15 mL conical tube and centrifuged at 959 g for 25 minutes at room temperature. After centrifugation, the PBMC layer was carefully collected and washed three times at room temperature with sterile phosphate buffer saline (PBS) (PBS; 137 mM NaCl, 2.7 mM KCl, 4.3 mM Na2HPO4·7H2O, and 1.4 mM KH2PO4, pH 7.4) at 327 g for 10 min. Cell viability was determined using 0.4% trypan blue exclusion, and viable cells were counted using Neubauer's hemocytometer.

Cell culture and dose-response determination

For the experimental treatments, 5x10^6^ viable Isolated PBMCs were maintained in RPMI 1640 medium supplemented with 10% fetal bovine serum and 0.71% of azithromycin solution. Cells were cultured in a humified atmosphere containing 5% CO_2_ at 37°C, and all of the following cell assays were maintained in the same conditions. To study the effect of glyphosate, both as pure salts and in the commercial formulation Faena®, PBMCs were treated with varying concentrations of glyphosate salts (200, 500, 1000, 2500, 3500, 4000 μg/mL) and Faena ® (1, 5, 15, 30, 60, 100 μg/mL) for 24, 48 and 72 hours. Each condition was analyzed in triplicate, and all experiments were repeated at least twice for reproducibility. Cell viability was assessed using trypan blue exclusion staining, and a dose-response curve was performed to estimate the median inhibitory concentration (IC50) using the Trimmed Spearman-Karber method [15].

Determination of the effect of *Bromelia pinguin* extract on PBMCs

The aqueous extract of *Bromelia pinguin* was prepared from freshly collected fruits collected in El Fuerte, Sinaloa by “Sinaloa’ s Autonomous University Biomedical sciences faculty research group”. Concentrations (1, 5, 15, 30, 60 µg/mL) were tested on PBMCs along with a control (water) and high concentration (135 µg/mL) group. Cells were exposed to the extract for 24, 48, and 72 hours and cell viability was assessed using trypan blue exclusion staining.

Co-exposure experiments

For the co-exposure assay, 5x10^6^ viable isolated PBMCs were treated with the closest concentration of the IC_50_ obtained from the dose-response curve for glyphosate salts (500 µg/mL) or Faena® (5 µg/mL) combined with *Bromelia pinguin* (60 µg/mL) extract. Cell viability was measured after 24, 48, and 72 hours for all groups.

Protein expression analysis by Western blotting

Protein Extraction and Quantification

Proteins involved in inflammation and apoptosis were assessed by Western blot. After treatments, cell cultures were centrifuged at 313 g for 5 minutes at 4°C, the supernatant was discarded, and pellets were washed with 1 mL of sterile PBS. This process was performed twice. Then 250 μL of Radioimmunoprecipitation Assay (RIPA) buffer was added, and samples were incubated on ice and vortexed every 10 minutes for 30 seconds. Samples were then centrifuged at 13,226 g for 10 minutes at 4°C, and the supernatant containing total proteins was collected and stored at -70°C. The samples were then transferred to the Faculty of Medicine at the National Autonomous University of Mexico for Western blot analysis. Protein quantifications were determined using the Bradford assay using bovine serum albumin (BSA) to generate a standard curve. Absorbance was measured at 595 nm using a Multiskan FC microplates photometer (Thermofisher ^TM^).

Determination of Inflammation and Apoptosis Markers by Western Blotting

Protein extracts from treated PBMCs were analyzed by Western blot to detect markers of inflammation and apoptosis. Equal amounts of protein (20 μL of sample mixed with 10 μL of loading buffer) were loaded onto SDS-PAGE gels, along with 2.5 μL of a protein ladder marker (PageRuler^TM^ Prestained Protein Ladder, Thermoscientific®). The Gels was run at 50 mA and 85 volts for 2 hours.

After electrophoresis, proteins were blocked with Tris-buffered saline (TBS) Tween 20 with 5% skimmed milk powder to reduce non-specific binding. Primary antibodies specific for Caspase-1, NLRP3, and PAPR-1 were applied at a 1:1000 dilution in TBS-T with 5% milk. Actin was used as a loading control, and cisplatin (250 μM) treatment was included as an apoptosis control. Secondary anti-rabbit antibodies were used at a 1:3000 dilution in the same blocking solution. Protein expression was detected and quantified by densitometric analysis using Image J 1.54 software.

Ethical considerations

Written informed consent was obtained from all participants. The study was approved by the Bioethics Committee of the Universidad Autónoma de Occidente in Los Mochis, Sinaloa, México (approval no. 28.10/2021) and adhered to the Declaration of Helsinki.

Statistical analysis

Statistical analysis was performed with IBM using SPSS Statistics for Windows, Version 26. Following the determination of data distribution, one and two-way ANOVA followed by post hoc tests (Tukey and Bonferroni) was used to identify differences between individual groups. P values less than 0.05 (p<0.05) were considered as significant.

## Results

PBMCs dose-response curves to glyphosate treatments and IC_50_ determination

**Figure 1:**
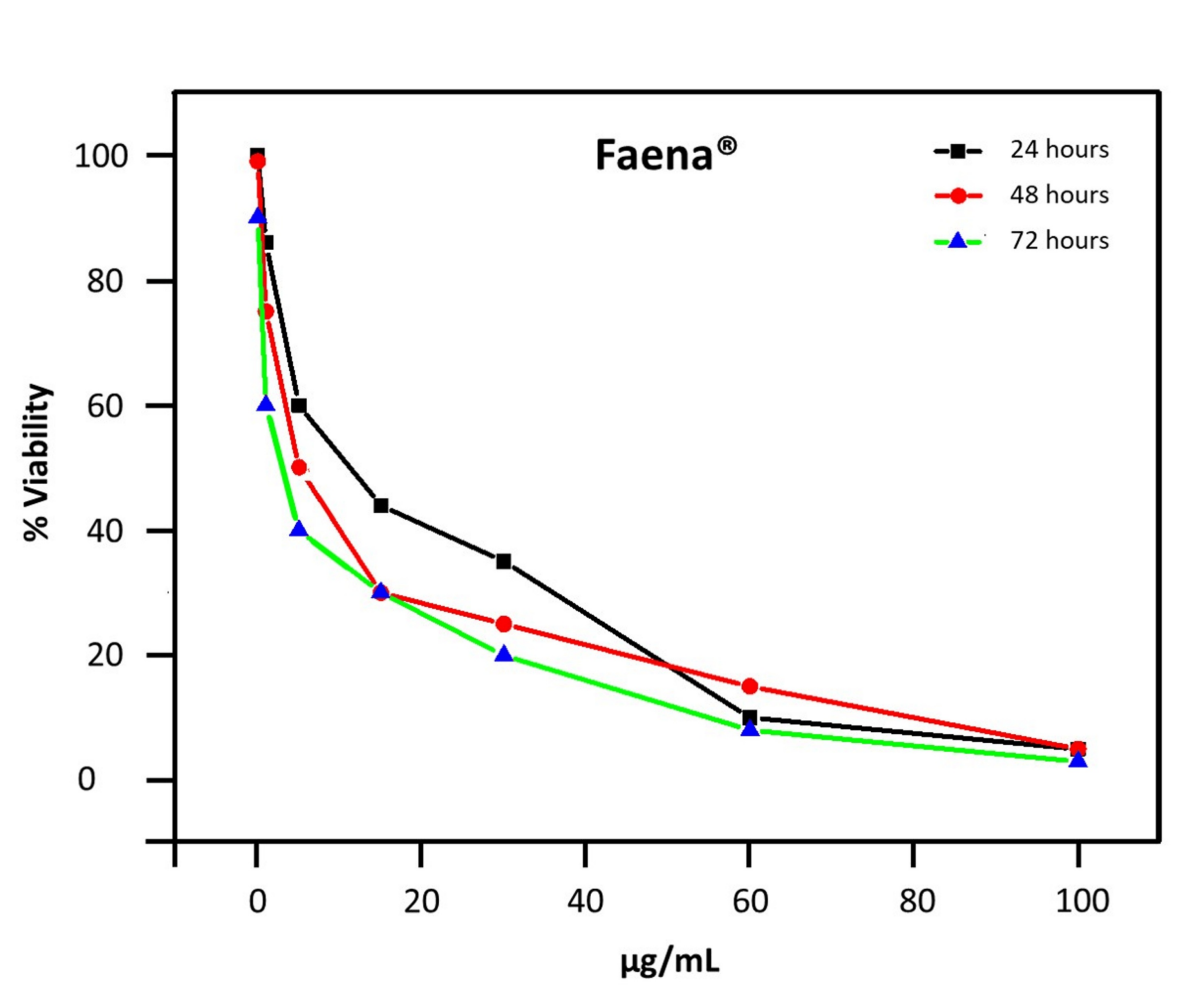
Viability of PBMCs following exposure to increasing concentrations of Faena®. The graph indicates viability percentages of PBMCs after 24, 48, and 72 hours of exposure to varying concentrations of Faena®. Cell viability percentages demonstrate a dose-dependent cytotoxic effect over time.

**Figure 2:**
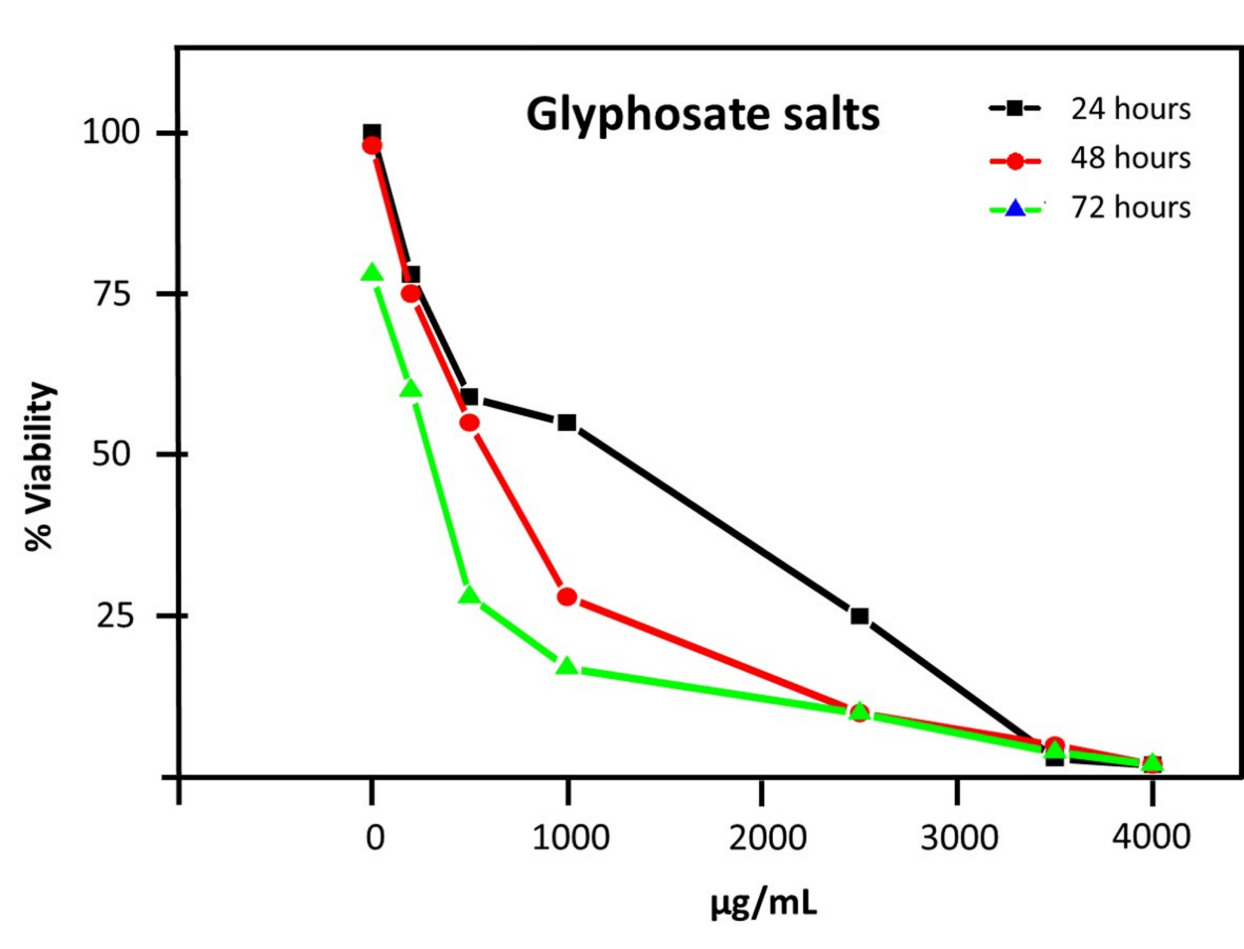
Viability of PBMCs following exposure to increasing concentrations of glyphosate salt. Graph indicates viability percentages of PBMCs after 24, 48, and 72 hours of exposure to varying concentrations of glyphosate salts. Cell viability percentages demonstrate a dose-dependent cytotoxic effect over time.

PBMCs exhibited a dose-dependent decrease in cell viability across 24, 48, and 72-hour assays when exposed to Faena® (Figure 1) or to glyphosate salts (Figure 2). Faena® reduced cell viability progressively over time, with IC_50_ values decreasing from 6.555 µg/mL at 24 hours to 3.5 µg/mL at 48 hours and 1 µg/mL for 72 hours.

Similarly, treatment with glyphosate salts led to notable reductions in cell viability, with IC_50_ values decreasing from 669.376 µg/mL at 24 hours, 350 µg/mL at 48 hours, and 100 µg/mL for 72 hours (Table 1). 

**Table 1:** Dose-dependent effects of Faena® and glyphosate salts on PBMCs viability at 24, 48, and 72 hours. IC_50_ values (µg/mL) indicate the concentration required for each herbicide treatment required to achieve a 50% reduction in cell viability at each time point.

Treatment	Time (hours)	IC_50_ (µg/mL)
Faena®	24	6.555
	48	3.5
	72	1
Glyphosate salts	24	669.376
	48	350
	72	100

Effect of Bromelia* pinguin* extract on PBMCs

Exposure of PBMCs to *Bromelia pinguin* extract alone (1-135 µg/mL) did not result in any significant reduction in cell viability at any concentration tested. Even at the highest concentrations (60-135 µg/mL) no cytotoxic effects were observed, although cell clustering was noted.

Co-exposure of PBMCs with *Bromelia pinguin*, glyphosate salts, and commercially available formulation Faena®

**Figure 3:**
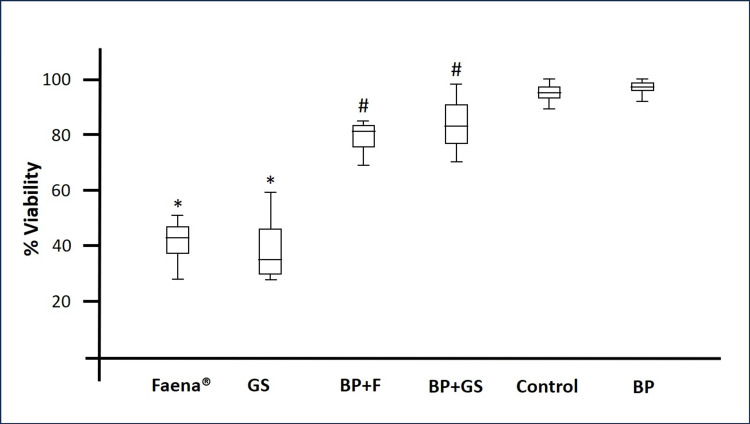
Percentage of PBMCs viability under different treatment conditions. The graph represents the viability of PBMCs subjected to various treatments at three-time points (24, 48, and 72 hours). Treatment groups include GS: Glyphosate salts, BP+F: *Bromelia pinguin* extract combined with Faena®,  BP+GS: *Bromelia pinguin* extract combined with glyphosate salts,  BP: *Bromelia pinguin* extract. * p<0.0001 compared to control group; #p<0.0001 compared to the respective herbicide treatment (GS or Faena®).

Co-exposure of PBMCs to glyphosate salts or Faena® combined with* Bromelia pinguin* extract significantly improved cell viability compared to herbicide-only treatments. In Faena® treatments, cell viability increased to approximately 70% at 24 hours and up to 80% at 48 and 72 hours of co-exposure. Similarly, in glyphosate salt treatments, cell viability increased to 70% at 24 hours, 85% at 48 hours, and more than 90% at 72 hours of co-exposure (Table 2 and Figure 3).

**Table 2:** Effect of co-exposure treatments on PBMCs viability. Table indicates PBMCs viability over 24, 48 and 72 hour periods of co-exposure treatments with *Bromelia pinguin* extract combined with Faena® or glyphosate salts.

Co-exposure treatments	Time (hours)	Cell viability (%)
Faena® + *Bromelia pinguin*	24	70%
	48	80%
	72	80%
Glyphosate salts + *Bromelia pinguin*	24	70%
	48	85%
	72	>90%

Western blot analysis of inflammatory and apoptotic markers

PBMCs treated with glyphosate salts, Faena®, *Bromelia pinguin* extract alone, and co-exposure with glyphosate formulations were analyzed by western blot for the expression of PARP-1, NRLP-3, and caspase-1 proteins.

PARP-1 Expression

**Figure 4:**
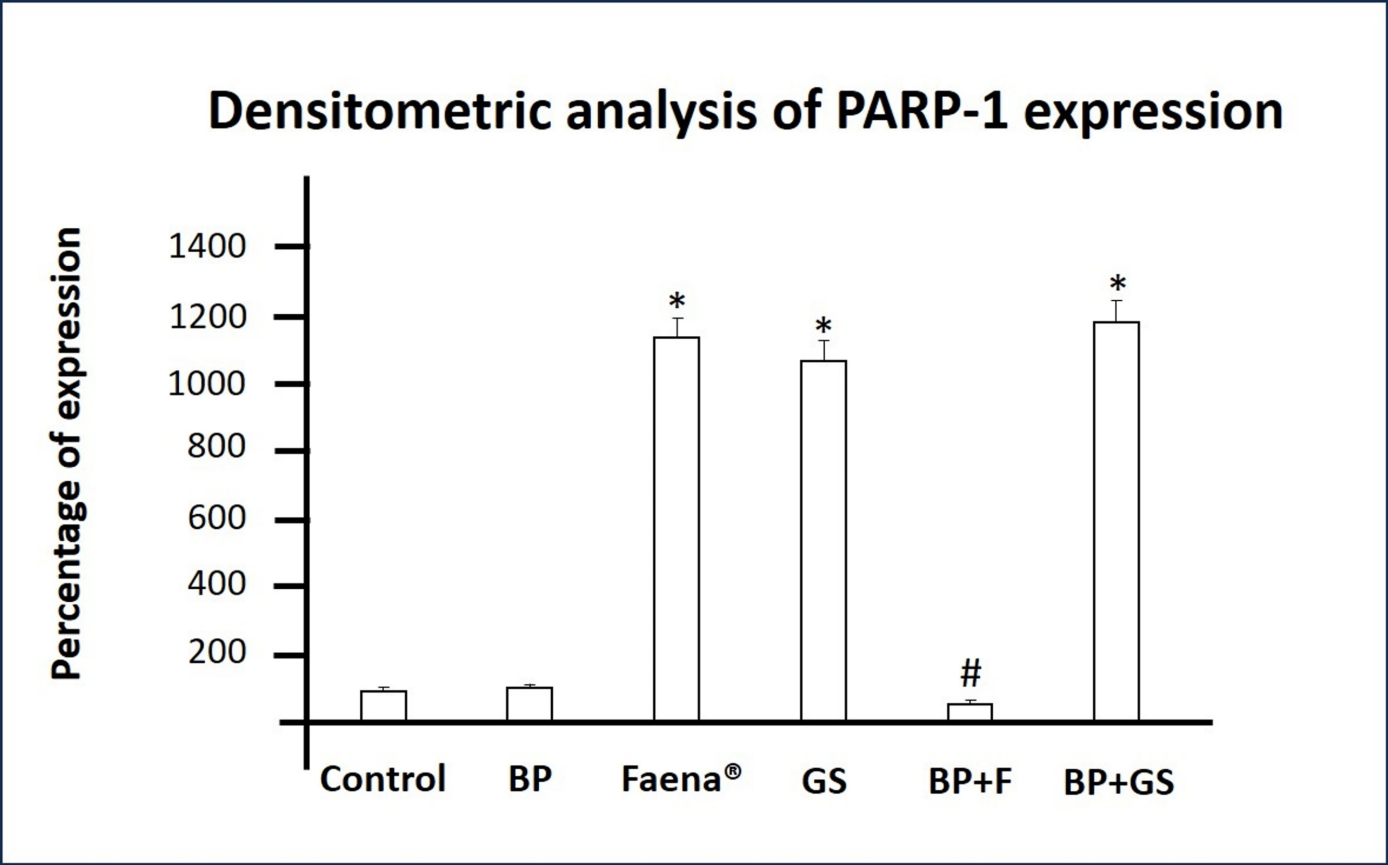
Densitometric analysis of PARP-1 protein levels. The graph indicates PARP-1 expression across different treatments. BP: *Bromelia pinguin* extract, GS: glyphosate salts, BP+F: *Bromelia pinguin* extract combined with Faena®, BP+GS: *Bromelia pinguin* extract combined with glyphosate salts. Densitometric values are normalized to the control group and set at 100 %. Values ​​are expressed as means±S.D. *p<0.0001 compared to control group; #p<0.0001 compared to Faena® group.

PBMCs treated with *Bromelia pinguin* extract exhibited PARP-1 protein levels similar to those of the control group. In sharp contrast, treatment with Faena® or glyphosate salts formulations resulted in a substantial increase in PARP-1 protein levels compared to the control group. Co-exposure of Bromelia pinguin extract with glyphosate salts did not reduce PARP-1 levels as they remained elevated and similar to glyphosate salt treatment alone. However, the Faena® co-exposure group exhibited a sharp decrease in PARP-1 levels, returning to levels similar to the control and *Bromelia pinguin *extract groups (Table 3 and Figure 4).

**Table 3:** Effects of Bromelia pinguin extract and glyphosate on PARP-1 expression in PBMCs. ns: nonsignificant, p≥0.05; ^a ^compared with control group; ^b ^compared to glyphosate salts group; ^c ^compared to Faena® group.

PBMCs treatment	p-value
*Bromelia pinguin* extract	ns^a^
Faena®	p<0.0001^a^
Glyphosate salts	p<0.0001^a^
Glyphosate salts + *Bromelia pinguin* extract	p<0.0001^a ^
	ns^b^
Faena® + *Bromelia pinguin* extract	ns^a^
	p<0.0001^c^

NLRP3 Inflammasome Expression

**Figure 5:**
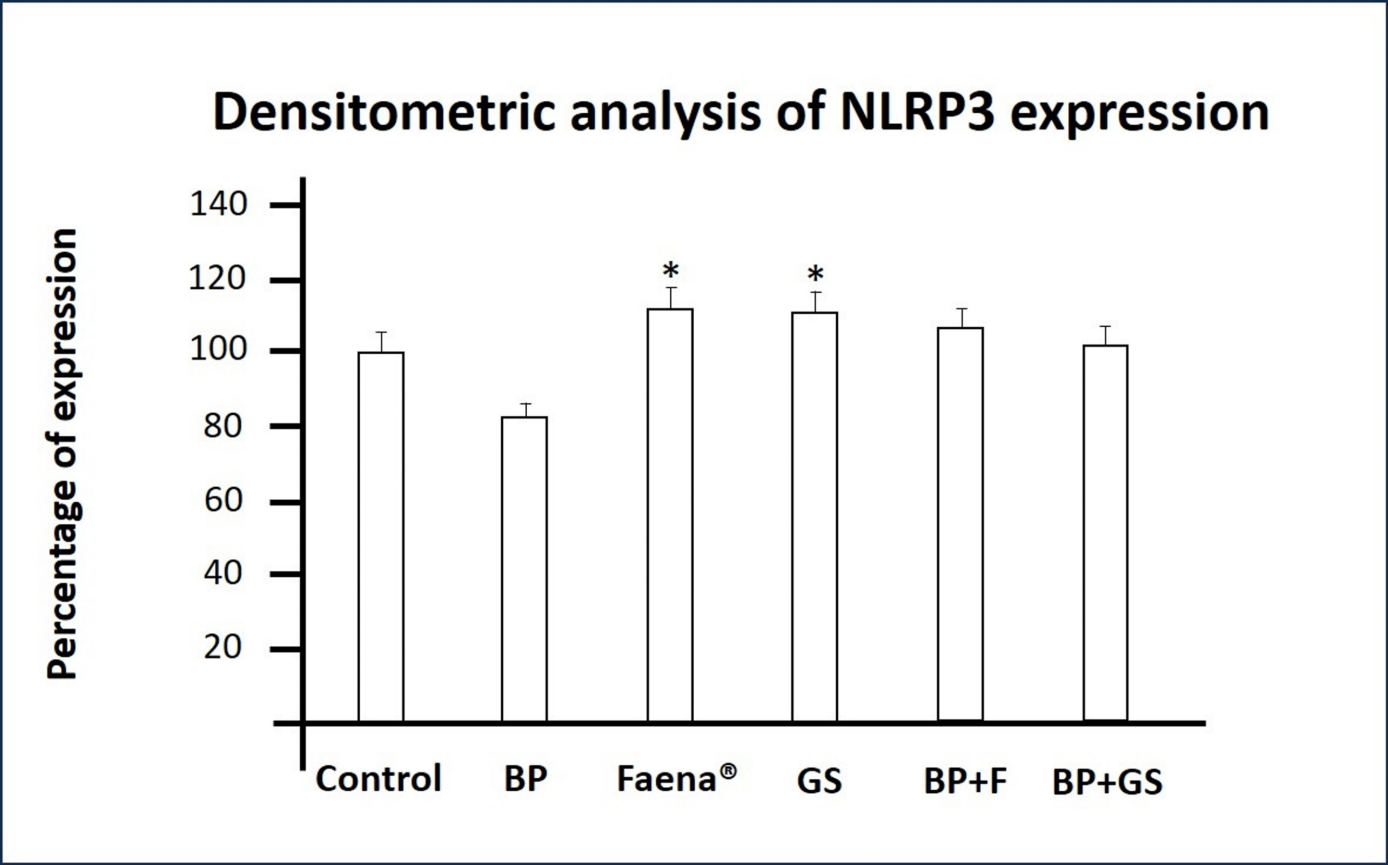
Densitometric analysis of NLRP-3 protein levels. The graph indicates NLRP-3 expression across different treatments. BP: *Bromelia pinguin* extract, GS: glyphosate salts, BP+F: *Bromelia pinguin* extract combined with Faena®, BP+GS: *Bromelia pinguin* extract combined with glyphosate salts.  Densitometric values are normalized to the control group, set at 100 %. Values ​​are expressed as means±SD *p<0.0001 compared to control.

PBMCs treated with *Bromelia pinguin* extract showed a significant decrease in NLRP3 levels compared to the control group. In contrast, glyphosate-treated groups showed a significant increase in NLRP3 protein expression compared to the control group. Interestingly, in co-exposure assays, NLRP3 levels in both glyphosate-treated groups returned to levels similar to those in the control group, indicating that the extract mitigated the herbicide-induced upregulation of NLRP3 (Table 5 and Figure 5).

**Table 4:** Effects of Bromelia pinguin extract and glyphosate on NLRP3 expression in PBMCs. ns: not significant; ^a ^compared with control group; ^b ^compared to glyphosate salts group; ^c ^compared to Faena® group.

PBMCs Treatment	p-value
*Bromelia pinguin* extract	p<0.0001^a^
Glyphosate salts	p<0.0001^a^
Faena®	p<0.0001^a^
Glyphosate salts + *Bromelia pinguin* extract	ns^a,b^
Faena® + *Bromelia pinguin* extract	ns^a,c^

Caspase-1 Expression

**Figure 6:**
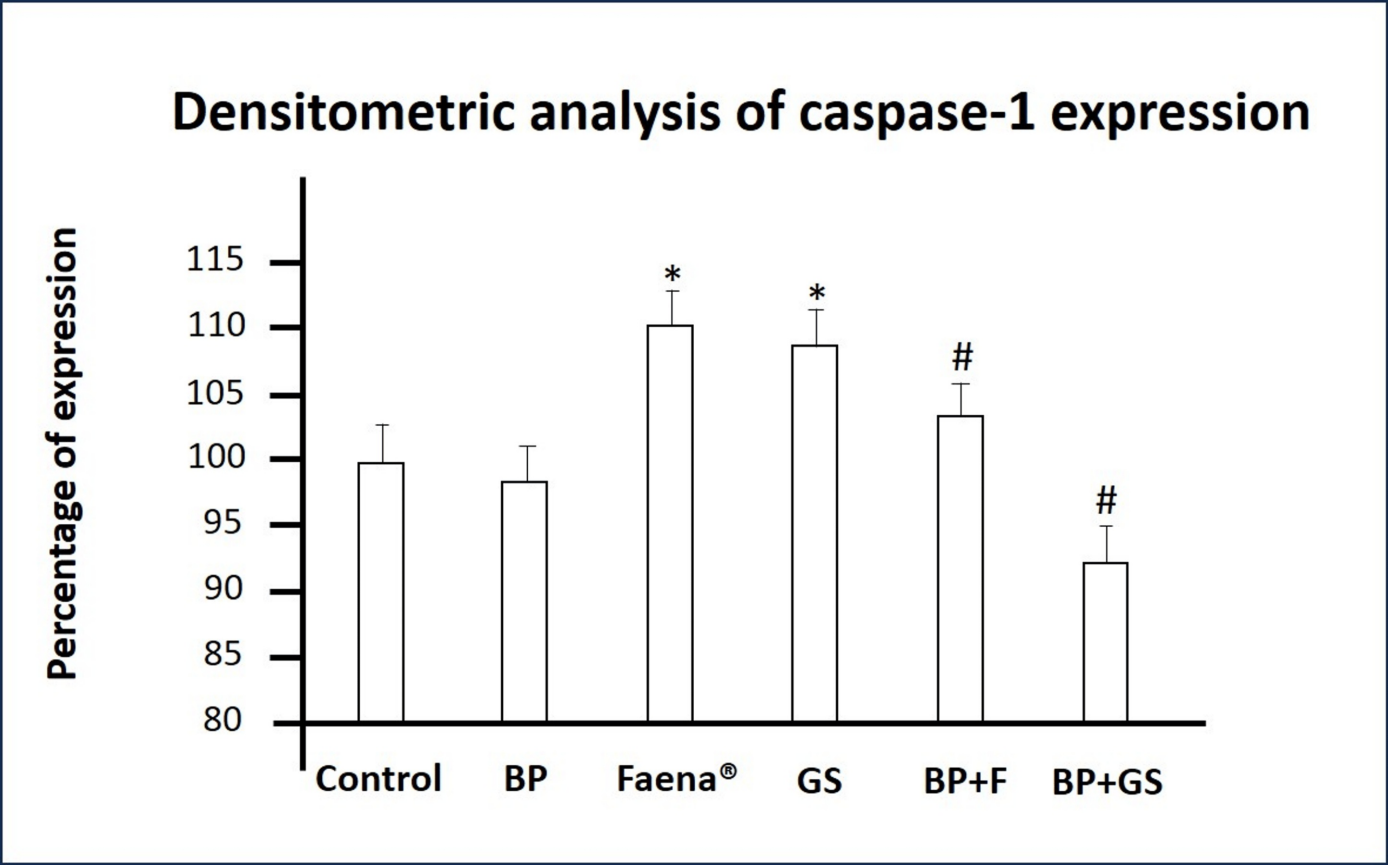
Densitometric analysis of Caspase-1 protein levels. The graph indicates Caspase-1 expression across different treatments. BP: *Bromelia pinguin* extract, GS: glyphosate salts, BP+F: *Bromelia pinguin *extract combined with Faena®, BP+GS: *Bromelia pinguin* extract combined with glyphosate salts. Densitometric values are normalized to the control group, set at 100 %. Values ​​are expressed as means±SD *p<0.0001 compared to control; #p<0.0001 compared to the respective herbicide treatment (GS or Faena®).

PBMCs treated with the *Bromelia pinguin* extract showed Caspase-1 levels similar to the control group. However, treatments with both glyphosate formulations resulted in a significant increase in Caspase-1 expression compared to the control group. In co-exposure assays, both Faena® and glyphosate salt groups treated with the extract showed significant Caspase-1 decreases compared to cells treated with the herbicides alone (Table 5 and Figure 6). 

**Table 5:** Effects of Bromelia pinguin extract and glyphosate on Caspase-1 expression in PBMCs. ns: not significant; ^a^ compared with control group; ^b^ compared to glyphosate salts group; ^c^ compared to Faena® group.

PBMCs Treatment	p-value
*Bromelia pinguin* extract	ns^a^
Faena®	p<0.0001^a^
Glyphosate salts	p<0.0001^a^
Glyphosate salts + *Bromelia pinguin *extract	p<0.0001^a^
	p<0.0001^b^
Faena® + *Bromelia pinguin* extract	p<0.0001^a ^
	p<0.0001^c^

## Discussion

In this study, we demonstrated for the first time the cytoprotective effect of *Bromelia pinguin* extract on human peripheral blood mononuclear cells when exposed to glyphosate salt or Faena®, a commercially available glyphosate formulation ready to use. Our results revealed that while glyphosate treatments induced significant cytotoxicity and increased levels of inflammatory and apoptotic proteins such as PARP-1, Caspase-1, and NLRP3, *Bromelia pinguin* extract significantly mitigated these detrimental cellular events, reducing protein expression to near-control levels and decreasing cell death.

We observed a dose-dependent cytotoxic effect in PBMCs exposed to both glyphosate treatments, with the commercial formulation being significantly more toxic than the pure salts. These results are consistent with previous reports demonstrating dose-dependent glyphosate toxicity in different human cell lines, with commercial formulations generally showing higher cytotoxicity [6]. Adjuvants contained in commercial glyphosate formulations can increase herbicide activity, however they can also increase glyphosate cellular uptake [6], which could explain the higher cytotoxicity we observed with Faena® formulation compared to pure glyphosate salts. In contrast, co-exposure to the aqueous extract of *Bromelia pinguin* improved cell viability and mitigated the upregulation of apoptotic and inflammatory markers Caspase-1, NLRP3, and PARP-1. Our findings add to the growing literature supporting the anti-inflammatory effects of Bromeliaceae plant extracts [11]. For example, *Bromelia hieronymi*, another Bromeliaceae family member, showed promising anti-inflammatory effects by inhibiting neutrophil migration and infiltration and reducing both acute and chronic inflammation in rat models [11]. Similarly, Bromelain, a protease mixture from Ananas comosus, has demonstrated similar anti-inflammatory properties in human and murine models by reducing neutrophil migration [12]. The proteolytic activity of these Bromeliaceae extracts likely contributes to their anti-inflammatory effects [11,12]. In our study, the observed anti-inflammatory mechanisms could be in part attributed to proteases as Bromelia pingüin is a known source of such bioactive compounds [16].

Western blot analysis provided important insights into the damage caused by glyphosate treatments in PBMCs and the protective mechanism of Bromelia pinguin that mitigates inflammatory and apoptotic markers associated with glyphosate exposure. When PBMCs were exposed to glyphosate, we noted an increase in the expression of NLRP3, PARP-1, and Caspase-1, which are crucial components of the inflammasome and apoptosis pathways. NLRP3 is an intracellular pattern recognition receptor protein that acts like a sensor of cell damage, danger signals such as damage-associated molecular patterns (DAMPs), and cellular stress, including reactive oxygen species. Once activated, it induces the formation of the NLRP3 inflammasome, which recruits the effector protein Caspase-1 [17,18]. Then, Caspase-1 promotes the maturation of the highly proinflammatory cytokines IL-1β and IL-18, which can lead to pyroptosis, a highly inflammatory lytic type of programmed cell death [17,18]. This type of cell death can release DAMPs, which can be detected by NLRP3, further increasing the inflammatory response [18]. In parallel, glyphosate formulations can induce oxidative stress and DNA damage [19], which can activate the DNA-repairing enzyme PARP-1 [20]. However, overactivation of PARP-1 during genotoxic and oxidative stress can lead to uncontrolled inflammation, generation of additional reactive oxygen species, and depletion of intracellular energy that could lead to necrotic cell death [21,22]. Together, these results indicate that glyphosate initiates inflammatory responses through NLRP3 inflammasome and promotes apoptosis through PARP-1 and Caspase-1 activation, which could also act as an inflammatory cell death loop.

Co-treatments of PBMCs with glyphosate and *Bromelia pinguin *extract significantly improved cell viability and reduced key apoptotic and inflammatory markers Caspase-1, NLRP3, and PARP-1. This effect was particularly marked in Faena® co-treated groups, where PARP-1 expression reached near control levels, suggesting that *Bromelia pinguin* extract could help mitigate herbicide-induced DNA damage. Bromelia pingüin contains bioactive compounds, such as diterpenoids and flavonoids [23], with antioxidant properties that could help mitigate oxidative damage. Flavonoids are also described to have potent anti-inflammatory properties [24], which might explain the observed reduction in NLRP3 inflammasome activation, thereby decreasing Caspase-1 activation. Additionally, antioxidants may help prevent damage to proteins, lipids, and DNA in PBMCs, leading to less DNA damage and, consequently, reduced Caspase-1 activation.

Glyphosate-based herbicides are the most used agrochemicals globally, and both occupational exposure and proximity to agricultural areas increase the risk of glyphosate toxicity [2,4,5]. This study was conducted In Sinaloa, México, a region with vast vegetation that includes endemic plants such as *Bromelia pinguin*, but also with heavy agricultural activities. Here, many persistent organic compounds, including pesticides and herbicides, have been detected in human samples, raising concerns about health risks for populations with sustained exposure, which may begin even before birth [25,26]. In this context, our findings of the potential of Bromelia pingüin to mitigate glyphosate toxicity are particularly relevant, as they suggest a promising therapeutic intervention. Additionally, the absence of toxicity in *Bromelia pinguin,* even at high concentrations, supports the plant's safety profile.

This study demonstrates the cytoprotective effects of *Bromelia pinguin* extract on PBMCs exposed to glyphosate, however it has several limitations. As an in vitro study, the findings may not fully replicate in vivo conditions, thus future studies in animal models or clinical settings are needed to confirm the effects. Additionally, only one glyphosate formulation (Faena®) was evaluated, and results may vary with other commercial products as adjuvants may increase herbicide efficiency and cytotoxicity. Our focus on short-term exposure left the long-term protective effects of *Bromelia pinguin* unexplored; thus, further investigations are needed. Finally, while the extract shows promise, the specific bioactive compounds responsible for its antioxidant and anti-inflammatory actions were not identified. Identifying these compounds could provide deeper insights into their protective mechanisms and more targeted strategies. Long-term effects of Bromelia pingüin, as well as its anti-inflammatory and cytoprotective effects on other cell lines and in combination with different herbicides and pesticides, should also be explored. This study provides a first approach toward the protective effect of Bromelia pingüin extract against glyphosate-based herbicides. Further studies should consider employing probability sampling methods and increasing cohort size to fully assess potential confounders.

## Conclusions

In this study we demonstrate that *Bromelia pinguin* extract effectively mitigates the cytotoxic effects of glyphosate and FAENA® on human PBMCs. The extract enhances cell viability and reduces the expression of key apoptotic and inflammatory markers NLRP3, Caspase-1, and PARP-1, suggesting a protective role against herbicide-induced damage. Future studies should investigate its potential clinical use as a co-treatment in populations exposed to glyphosate herbicides.
